# Supplementary material for: Are carbon emissions trading and green financial instruments synergistic? -Comprehensive quantitative research based on content analysis
Source: PLoS One. 2024 Mar 7;19(3):e0298601. doi: 10.1371/journal.pone.0298601 (PMC10919601; doi:10.1371/journal.pone.0298601)
Supplement: S1 Data — (ZIP) [file pone.0298601.s001.zip › Data/地级市Z_F工作报告.zip!/╜¡╦╒/╠⌐╓▌╩╨/└┤╫╘╣1⁄2╓┌║┼í╛┬φ┐╦╩2╛▌═°í┐_2003.docx]

各位代表：

现在，我代表第一届市人民政府向大会作工作报告，请予审议，并请市政协各位委员和其他列席会议人员提出意见。

六年多来的工作回顾

市一届人大一次会议以来，我们在中共泰州市委领导下，以邓小平理论和“三个代表”重要思想为指导，积极贯彻省委、省政府组建地级泰州市的战略意图，认真落实市一届人大历次会议确定的目标任务，团结拼搏，艰苦创业，抢抓机遇，开拓进取，全市经济实力显著增强，社会事业协调发展，人民生活明显改善。

国民经济持续稳定发展，人民生活水平不断提高。2002年全市国内生产总值突破500亿元，达504.26亿元，比1996年增长85%，年均增长10.8%。人均国内生产总值突破万元，达10018元，比1996年增加4093元。三次产业结构由1996年的21：45：34调整为14：50：36。扎实推进农业结构战略性调整，多种经营产值占农业总产值的比重逐年提高，农业产业化经营有了良好开端。组织开展“学春兰”活动，突出抓好重点企业培育、重点项目实施和重点产品开发，工业经济发展速度和运行质态不断提高。全市工业增加值年均增长12%。建筑业持续快速发展，施工产值由建市之初的全省第5位上升到第2位，2家企业获得国家特一级资质。认真抓好服务业重点项目和新兴行业，服务业增加值占国内生产总值的比重提高2个百分点。2002年全市财政收入53.1亿元，比1996年增长 1.6 倍，年均增长16.9%。城镇居民人均可支配收入7788元，农民人均纯收入3834元，城乡居民储蓄存款余额336亿元，分别比1996年增长43.6%、29.9%和121.1%。

改革不断向纵深推进，经济发展的动力和活力进一步增强。大力推进市（区）属工贸企业“三置换一保障”（置换公有产权、职工身份和土地使用权，实行社会保障）改革，改制面达63%。扎实抓好乡镇企业产权制度改革，改制面达99.8%，集体资本占企业实收资本的比重降至10%。鼓励、支持和引导私营个体经济发展，2002年末全市私营个体企业注册资本103.6亿元，为1996年末的9.5倍，完成税收为1996年的4.9倍。全面推进农村税费改革，改革后农民合同内负担减轻30.4%。认真做好就业再就业和社会保障工作，六年多来有近7万名下岗职工实现了再就业，“三条保障线”政策得到落实。按照上级部署，实施了市县乡政府机构改革。财税、金融、粮食、供销、外贸以及社会事业等领域的改革也取得了积极进展。

对外开放进一步扩大，经济国际化水平逐步提高。全市累计完成合同协议利用外资12.93亿美元，实际利用外资7.27亿美元，年均增长17.5%和15%，其中2002年完成5.05亿美元和2.1亿美元，分别比上年增长97.5%和74%。累计完成自营出口17.46亿美元，年均增长21.8%，其中2002完成4.2亿美元，比上年增长9%。春兰、汇丰等企业相继在境外投资办厂，境外工程承包和劳务输出势头良好。开发区建设取得明显进展，2002年5个省级经济开发区合同协议利用外资和实际利用外资分别比上年增长139.9%和70.7%。泰州海关、出入境检验检疫局、海事处等口岸查验机构相继设立。积极开展对外交往，先后与4个外国城市结为友好城市。对台和侨务工作进一步加强。

中心城市面貌大变，基础设施建设取得突破性进展。中心城市围绕“五年面貌大变”的目标和“建设大城市、塑造新形象”的要求，累计完成城建投资90多亿元，建成区面积由不足20平方公里扩大到40多平方公里。各市市区和重点中心镇建设取得长足发展，建成12个省级新型小城镇。全市城市化率由1996年的29.3%上升到2002年的44.9%。强化城市管理，环境质量逐步提高。组织实施交通建设“黄桥战役”，建成宁通、广靖、宁靖盐高速公路泰州段和泰高公路、兴泰公路、328国道泰姜段等一批干线公路、新长铁路泰州段以及泰州港3座万吨级以上码头。完成了长江引水工程、泰州引江河工程、泰东河新通扬运河段工程、“四•三”水利工程（用3年时间基本完成江堤达标工程、里下河无坝市工程、通南地区节水灌溉工程、严重淤浅河道疏浚工程）和部分城市防洪急办工程。全面进行了城市和农村电网改造，重点建设了电信网、移动通信网，建成开通了广电宽带数据业务网。全市固定资产投资累计完成758亿元，年均增长17.1%。

社会事业协调发展，精神文明建设迈出新步伐。认真实施科技“四大工程”（人才、挂靠、创办、开发工程），人才资源总量从建市之初的全省第12位上升到第7位，科技对工业经济增长的贡献率提升为全省第5位。大力发展教育事业，积极推进素质教育。建成了职业技术学院等3所大专院校，现在校学生达1.6万人。创建了一批国家示范高中和省级重点高中。扎实抓好农村义务教育管理体制改革和中小学布局调整，薄弱学校和危房改造取得显著成绩，普及九年制义务教育成果得到巩固和发展。重视文化、广播电视、卫生、体育和计划生育工作，建成3个省级文化先进市、4个初级卫生保健先进市和3个计划生育先进市、1个计划生育模范市。广播电视事业发展迅速，全市有线电视通村率达90%，城乡综合入户率达40%。改水改厕成效显著，全民健身运动和竞技体育取得了积极进展。土地管理和环境保护得到加强。民政、统计、民族宗教、旅游、地方志、档案、科普、人防、气象和老龄、妇女儿童、未成年人保护、残疾人工作有了新的进展。以提高市民素质和城市文明程度为目标，不断加强精神文明建设。积极开展群众性文明创建活动，市区和泰兴市被命名为省级文明城市。双拥工作不断加强，人民武装工作取得长足进步，征兵工作连续6年受到省政府表彰。

民主法制建设取得新进展，政府自身建设得到加强。自觉接受市人大及其常委会的法律监督、工作监督和市政协的民主监督，重视和发挥各民主党派、工商联的参政议政作用，认真办理市人大代表建议、政协委员提案。积极推进政务公开、村务公开和厂务公开，抓好村委会、居委会建设。重视信访、人民建议征集和民调工作。组织“三五”、“四五”普法，按照加入世贸组织的新要求，制定、清理和完善地方规范性文件。加强社会治安综合治理，持续开展“严打”斗争，取缔法轮功邪教组织，维护社会稳定。整顿市场经济秩序，净化市场环境。工商管理、审计监督、质量技术监督、药品质量管理、物价调控工作得到加强。扎实抓好政府自身建设。市政府及其组成部门领导班子“三讲”教育活动取得成效，公务员队伍建设得到加强。组织实施“421”工程，推进政府工作目标管理。率先推进行政审批制度改革，稳步推进干部人事制度和财政管理体制改革，对市级机关中层干部全面实行竞争上岗，积极推行部门预算和政府采购制度，加强预算外资金管理。认真落实党风廉政建设责任制，纠风、专项治理和查处违纪违法案件工作取得成效。

回顾过去，我们取得的成就令人鼓舞。在圆满完成市第一届人民代表大会确定的各项任务的今天，我谨代表第一届市政府，向积极投身于新泰州建设和发展的广大干部群众，向给予政府工作关心与支持的人大代表、政协委员，向各民主党派、工商联、无党派人士以及社会各界人士，向中央、省驻泰机构以及人民解放军驻泰部队指战员和武警官兵、公安干警，向所有关心支持泰州改革开放和现代化建设的海内外朋友们，致以崇高的敬意并表示衷心的感谢！

回首新泰州走过的历程，我们主要有以下几点体会：

必须突出“第一要务”，始终高扬加快发展的主旋律。发展是硬道理，也是全市500万人民的热切愿望。六年多来，我们立足于为新泰州的长远发展打基础、增后劲，始终把加快发展作为第一要务，坚持用发展的目标凝聚人心，用发展的办法解决问题，用发展的业绩评判得失，用发展的成果造福人民。突出加快经济发展，突出中心城市建设，突出提高人的素质，抢抓区划调整、江阴长江大桥通车、国家扩大内需和国际资本向长江三角洲集聚等机遇，全面实施市第一次党代会提出的科教兴市、开放开发、规模经济、城乡一体、可持续发展“五大战略”，实现了新泰州经济社会发展的良好开局。实践证明，加快发展是富民强市之本。只有聚精会神搞建设，一心一意谋发展，才能实现快速崛起，才能真正落实省委、省政府组建地级泰州市的战略意图。

必须坚持与时俱进，推进各项工作的创新。过去的六年是与时俱进、创新发展的六年。我们始终坚持以“三个有利于”为根本标准，大力推进以解放思想、加快发展为核心的观念创新，以企业产权制度改革为重点的制度创新，以科技“四大工程”为载体的技术创新，以城市经营和基础设施建设市场化运作为主要内容的投融资体制创新，以及以行政审批制度改革和软环境建设为突破口的行政管理创新，有力地推动了新泰州建设和发展。实践告诉我们，只有坚持解放思想、实事求是、与时俱进的思想路线，积极探索具有时代特征和泰州特色的发展新路，才能不断开创工作新局面。

必须弘扬泰州精神，始终保持良好的精神状态。事在人为，人在精神。保持奋发向上的精神状态，对于百事待举的新泰州，显得尤为重要。六年多来，我们和全市人民一道，大力弘扬和积极实践泰州精神，团结一致、齐心协力，艰苦奋斗、攻坚克难，自强不息、勇创一流，在宏观经济环境不够宽松的背景下实现了经济的较快增长，在对外开放基础相对薄弱的情况下保持了开放型经济的持续发展，在建设资金十分紧缺的条件下建成了一批社会事业和基础设施重点工程，在企业改革力度不断加大、经济运行中长期积累的深层次矛盾渐次暴露的环境下维护了社会稳定。实践证明，泰州精神是我们的宝贵精神财富，大力弘扬和不断升华泰州精神，必将为全市改革开放和现代化建设注入源源不断的动力。

必须实践根本宗旨，把维护和实现最广大人民的根本利益作为一切工作的出发点和归宿。贯彻“三个代表”重要思想，本质在坚持执政为民。六年多来，我们始终坚持党的群众路线，牢记人民利益高于一切，坚持通过深入基层、深入群众和办好市长公开电话、做好信访工作等途径来了解民意，通过每年为全市人民办20件实事、为市区人民办10件实事等方式来改善民生，通过开展市级机关挂钩扶持黄桥老区薄弱乡和机关干部结对帮扶城市贫困户活动、抓好“南户北村”帮扶工作（扶持黄桥老区贫困户脱贫致富、兴化部分经济薄弱村通村公路建设）以及建立重病患者救助、贫困家庭大学生入学资助制度等办法来排解民忧，通过推进政务公开、建立重大事项听证制度等措施来扩大民主，得到了人民群众的欢迎、拥护和支持。实践使我们深切体会到，只有全心全意为人民谋利益，在排解民忧的基础上凝聚民心，在关心民生的前提下使用民力，在扩大民主的过程中集中民智，发挥群众在改革开放中的主力军作用，我们才能不辱使命、有所作为，才能无愧于历史，无愧于人民。

六年多来，全市经济建设和社会事业虽然取得了长足进步，但仍存在一些不容忽视的问题，主要是：经济结构性矛盾依然突出，重大增长点不多，区域经济发展不平衡性较大；经济外向度还不高，投资环境有待进一步改善；城镇就业再就业矛盾比较突出，农民特别是纯农户收入增长缓慢；中心城市的辐射带动作用还不强，城乡基础设施还不够完善；精神文明和民主法制建设有待进一步加强；政府职能转变需要进一步加快，机关从管理型向服务型转变的深度不够，服务质量有待进一步提高等等。这些问题，需要通过进一步深化改革、创新思路、转变作风、狠抓落实，在今后的工作中认真加以解决。

今后五年的奋斗目标和主要任务

各位代表，今后五年是泰州全面建设宽裕型小康社会、向基本实现现代化迈进的关键时期。我们要以邓小平理论和“三个代表”重要思想为指导，全面贯彻落实党的十六大精神，围绕市第二次党代会提出的“富民强市，快速崛起”的目标，不断完善社会主义市场经济体制和机制，扎实推进经济结构战略性调整，大力实施科教兴市、大开放、城市化和可持续发展战略，协调推进三个文明建设，努力把泰州建设成为经济繁荣、人民富裕、社会文明、环境优美的新兴工贸港口城市，成为长江三角洲城市群中的后起之秀。到2007年，全市国内生产总值超过800亿元，人均1.6万元以上；财政收入力争突破100亿元；全社会固定资产投资累计1300亿元；实际利用外资累计达到25亿美元；城市化水平提高到52%；城镇居民人均可支配收入和农民人均纯收入分别为10000元和5000元左右。

抓好经济结构战略性调整，构筑产业发展新高地。加快农业和农村经济发展，累计引进外资、民资和股市资本30亿元投入农业，一大批新的农业企业成为市场竞争主体，农业区域特色初步形成，农产品检验检测体系基本建成，农业支持保障体系得到强化，农业增加值年均增长5%以上。农村劳动力五年新转移25万人以上，农民生活日渐宽裕。

走新型工业化道路，工业经济整体素质明显提高。工业“30强”企业资本营运、技术创新、国际经营、现代管理和抗御风险能力明显增强，2007年营业总收入达到1000亿元以上。机电、医药、化工、造船、纺织等传统产业在运用新技术改造的过程中不断壮大，制造业高地初具雏型。以新材料、新医药、新能源和信息业为重点的高新技术产业迅速发展，2007年高新技术产业增加值占工业增加值的比重提高到30%以上。建筑业在改革、调整中加快发展，经济总量位于全省前列。

推动服务业结构优化和产业升级，各类传统服务业在改造中提升层次，现代物流、信息服务、旅游、社区服务等新兴服务业在竞争中发展壮大，服务业社会化、市场化、国际化步伐加快，占国内生产总值的比重提高到40%以上。

发挥比较优势，区域共同发展的格局初步形成。靖江、泰兴、高港的沿江地区与苏南联动发展，形成初具规模的沿江产业带，基本建成沿江公路，积极推进泰州电厂和过江通道等重大项目建设。姜堰、兴化的里下河地区发挥资源优势，生态农业和私营工业形成规模，兴泰公路北延、沙钓公路等基础设施建成运营。泰兴、姜堰的黄桥老区发挥政策、人才等效应，初步形成特色农业、工业特色产业、建筑业发展优势，完成姜八线、如过线等干线公路改造。区域之间紧密合作，错位发展，在扩大开放中共同提高，在加快发展中缩小差距。

认真做好就业再就业和社会保障工作，把泰州建设成为全省就业较为充分的城市，并基本形成独立于企事业单位之外、资金来源多渠道、管理服务社会化，广覆盖、多层次的社会保障体系。

加快开放型经济和私营个体经济发展，培育两大经济增长点。适应加入WTO的新形势，全面提高经济国际化水平。招商引资取得突破，实际利用外资年均增长30%以上。5个省级经济开发区集聚、创新和产出功能明显增强，其中泰州经济开发区基本形成“三园联动”的格局，五年协议利用外资累计10亿美元，实际利用外资累计6亿美元，新增开发面积达20平方公里。出口持续快速增长，2007年全市自营出口达10亿美元。外经合作进一步扩大。港口开放功能趋于完善。全市投资环境明显优化，跻身长江三角洲最适宜投资和创业的城市行列。

加快私营个体经济发展。私营个体经济平等竞争的市场环境进一步形成，民间资本日益活跃，私营企业观念、体制、技术和管理创新步伐加快，一批乡镇企业园区初具规模。2007年，私营个体经济增加值占国内生产总值的比重提高到45%以上，对经济增长的贡献份额上升到55%以上。

推进体制创新和技术创新，增强发展的两大动力源。坚持不懈地推进改革，农村税费改革进一步深化，企事业单位改革取得突破。市（区）属工贸企业、经营性服务类事业单位“三置换一保障”改革和建筑企业产权制度改革全面完成，公有资本从一般竞争性领域基本退出，企业法人治理结构不断完善，初步建立充满活力的现代企业制度。

进一步加快技术创新，逐步健全以企业为主体的技术创新体系、中小企业的技术创新服务体系和多元化投融资体系。今后五年，工业技改投入累计500亿元以上，滚动开发3000万元以上规模的技改项目350个以上。人才高地建设取得较大进展，2007年全市人才资源总量突破30万人。

加强环境建设，形成区域发展的新平台。围绕“建设大城市，塑造新形象”的目标，坚持壮大工贸港口城市的产业基础与培育水城一体的城市特色并举，加快城市建设与加强城市经营并举，新区建设与旧城改造并举，做大、做强、做优、做美中心城市。经过五年努力，旧城焕然一新，新区展露新姿，高港中心区功能进一步完善，三大商业中心区全部形成，环城河风光带、凤凰河景观带建成开放，中心城市建成区面积扩大到60平方公里，进入大城市、国家环境保护模范市和省级园林城市行列。加快县级市城区和重点中心镇建设，4个县级市建成中等城市，市域城镇发展整体水平进一步提高。

继续加快城乡基础设施建设。基本建成城市防洪和长江、淮河流域性防洪排涝工程体系，农田水利基础设施明显改善。区域性供水工程取得阶段性成果，市区全部人口和姜堰、兴化的部分地区群众以及靖江、泰兴地区群众饮用上长江水。溧（阳）海（安）高速、宁启铁路泰州段等重点交通工程竣工，乡镇政府到高速公路半小时交通圈形成，全市高速公路网络化、干线公路等级化、乡村公路标准化，交通基础设施初步现代化目标基本实现。进一步完善信息、电力基础设施，承载能力明显增强。

加强软环境建设，政策、服务、法制和人文环境进一步改善。加快转变政府职能，基本形成行为规范、运转协调、公正透明、廉洁高效的行政管理体制，建立公平公正、竞争有序的市场环境。

坚持物质文明、政治文明、精神文明协调推进，发展社会主义民主政治和先进文化。进一步扩大基层民主，人民群众依法直接行使民主权利。加强法制建设，依法治市水平明显提高，全市政治稳定，社会安定。深入开展群众性精神文明创建活动，市区进入全国文明城市创建工作先进市行列，四市进入省级文明城市行列。优先发展教育事业，整合优化教育资源，基本普及高中阶段教育，力争建成本科层次的高校。继续加快文化、广播电视、卫生、体育等社会事业的改革和建设，建成群艺馆、图书馆新馆、体育中心等一批新的公益设施，市人民医院达到三级甲等医院标准。进一步实施可持续发展战略，人口、资源、环境协调发展。

各位代表，经过五年努力，我们要把泰州基本建成宽裕型小康社会，基本形成符合社会主义市场经济规律的体制和机制，基本确立“一大四中”的城市格局，基本具备长江三角洲苏中一流的发展环境。我们坚信，只要全市人民同心同德，奋力拼搏，“四个基本”的目标就一定能够实现，泰州的明天一定更加美好！

2003年工作建议

今年是全面贯彻落实党的十六大精神的第一年，是实施“十五”规划承上启下的关键之年，又是新一届政府开局之年。做好今年的政府工作，意义十分重大。

今年政府工作的总体要求是：以邓小平理论和“三个代表”重要思想为指导，全面贯彻党的十六大精神，认真落实中央、省经济工作会议和市委二届四次全体（扩大）会议精神，突出发展主题，实施四大战略，改革要有新突破，开放要有新局面，私营个体经济要有新跨越，结构调整要有新进展，发展环境要有新改善，社会事业要有新发展，人民生活要有新提高，政治文明和精神文明建设要有新进步，向着全面建设小康社会的奋斗目标加速前进。

今年全市国民经济和社会发展主要调控目标建议安排为：国内生产总值增长11%以上，其中一产、二产、服务业增加值分别增长5%、13%以上和11%以上；财政收入增长13%；全社会固定资产投资增长13%；社会消费品零售总额增长9.5%；自营出口增长12%，实际利用外资增长50%；城镇居民人均可支配收入增长6%，农民人均纯收入增长5%；城镇登记失业率控制在4.5%以内;人口自然增长率控制在4‰以内。

以招商引资为主要抓手，实现开放型经济新跨越。紧紧抓住国际资本加快向长江三角洲地区集聚的重大机遇，集全民之智，举全市之力，推进招商引资。坚持境外资本、市外民资和股市资本一齐招，三次产业、社会事业、基础设施和城市建设招商引资一齐抓，市县乡三级联动，形成全方位、宽领域、多层次、立体化的招商引资格局。加强招商引资的主阵地建设，市和各市（区）重点抓好省级经济开发区建设，指导重点乡镇办好特色工业园区，鼓励工业规模企业建设专业园区，同时抓好市级重点农业园区的规划、建设和招商。大力支持泰州经济开发区建设和发展，切实改善投资环境，落实市级经济管理权限，努力保持其快速发展的良好势头。着力推进沿江大开发大开放，按照高起点开发、有序性开发、可持续性开发和跨江联动开发的指导原则，抓紧编制沿江产业布局、港口开发、基础设施建设总体规划，研究出台激励政策。率先启动靖江市与江阴市的联动开发，积极推进沿江整体合作开发。把人力资源作为招商引资第一资源，加强专业招商队伍建设。积极实施市场多元化、以质取胜和科技兴贸战略，千方百计扩大出口。支持外经企业开拓国际劳务和工程承包市场，鼓励和推动具有比较优势的企业“走出去”，开展境外投资。筹建泰州边防检查站，完善泰州港一类开放口岸功能。做好外事、侨务和对台工作，积极开展对外交流合作。

以优化创业环境为着力点，促进私营个体经济加快发展。抓住民资崛起的重大机遇，把营造良好的创业环境放到更为突出的位置，进一步放大戴南、张郭效应。依法保护私营个体经营者的合法权益，落实私营个体经济平等待遇，改善部门服务承诺工作。完善信用担保体系，扩大担保基金规模，建立服务便捷、收费低廉的抵押评估体系，推进金融工具创新，拓宽贷款流向私营个体经济的渠道。进一步保护和激发广大群众的创业热情，促使本地富人成为投资者、能人成为经营者、民间资本成为创业资本，形成新的投资潮、创业潮。引导私营企业增强危机意识和发展意识，推进技术创新，开展合资合作，提高管理水平，重视人才培养，加快规模扩张和素质提高。

以市（区）属工贸企业“三置换一保障”为重点，继续深化各项改革。坚持加快改制进度与提高改制质量的统一，着力打好市（区）属工贸企业“三置换一保障”改革攻坚战。进一步加大政府对企业改革的政策扶持力度，多渠道筹措改制成本，确保今年底85%以上的市（区）属工贸企业完成“三置换一保障”改革任务。坚持一企一策，多方案比选，注重引进民间资本、海外资本、外地资本参与市（区）属企业改革，建立起符合市场经济法则的运行机制和股权、经营者流动机制。严格依法办事，防止公有资产流失。按照社会化发展、市场化运作的方向，加快推进事业单位改革。按照政事分开的原则，对管理类事业单位进行清理，将没有法律法规授权和依法委托的行政职能收归政府部门，压缩机构和编制；按照资源优化的原则，对社会公益类事业单位合理调整布局，实行总量和结构控制；按照事企剥离和公有资本有序退出的原则，对经营性服务类事业单位进行“三置换一保障”改革，今年改制面达30%。加强国有资产营运体系建设，落实国有资产经营管理的各项措施，确保保值增值。继续深化农村税费改革，建立健全农民负担监督管理新机制。深化财政、金融体制改革，在市级机关全面推行部门预算，建立国库集中收付制度，完善政府采购体系和金融监管体系。进一步整顿和规范市场经济秩序，强化市场监管，营造公平竞争环境。增强全社会信用意识，加快建设“诚信泰州”。

以增加农民收入为核心，全面繁荣农村经济。以培植市场竞争主体为突破口，推进农业和农村经济结构的战略性调整。大力发展“三资”农业，广泛吸引民间资本、工商资本、外商资本参与农业开发，积极兴办农业“三资”企业。牢固确立致富农民必须减少农民、转移农民的思路，通过推进农村工业化、城镇化、城市现代化和经济外向化，消除体制和政策障碍，增加劳务输出，促进农村富余劳动力向非农产业和城镇转移。继续抓好农产品布局结构和品种结构调整，培育具有比较优势和发展潜力的特色产业。加强农产品质量标准体系、检验检测体系和认证体系建设，抓好无公害农产品基地建设。推进农产品超市经营、连锁配送、网上销售，拓宽农产品流通渠道。按照依法、自愿、有偿的原则，进行土地承包经营权流转，逐步发展规模经营。增加农业投入，重点支持农业结构调整、农产品质量建设、农业服务体系和农村生态环境建设。

以加速新型工业化为方向，促进工业结构优化升级。把培大育强骨干企业作为调整重点，通过组建服务小组，强化具体指导，落实扶持措施，着力培植全市工业经济30强。加快信息技术的推广应用，促进企业技术和管理升级。积极发展对经济增长有重要带动作用的高科技产业，突出抓好210个年新增销售3000万元以上重点新品的开发，确保70个新品达效。继续运用高新技术和先进适用技术改造传统产业，重点抓好110个投资3000万元以上重大技改项目的实施，确保竣工投产50项以上。以扬子江药业集团为龙头，加快沿江新医药产业带建设。全年技改投入确保增长15%。高度重视和扎实推进项目储备工作。进一步完善中小企业服务体系，继续支持中小企业向“专、精、特、新”方向发展。加强产品质量技术监督，引导企业采用国际和国内先进标准组织生产。强化扭亏脱困工作，努力降低亏损面和亏损额。发挥我市建筑业比较优势，抓住北京办奥、上海办博、西部大开发、城市扩容和“南水北调”带来的机遇，加大市场开拓力度。加快推进建筑企业改革，加强技术装备和质量管理，提高发展水平。

以发展现代服务业为突破口，进一步提高第三产业在国民经济中的比重。整合交通、港口、邮政等物流资源，加快发展现代物流业。把社区服务业作为重要增长点精心培育，充分挖掘其增长潜力。进一步搞活房地产二级市场，培育房屋租赁市场，推动住房消费。大力推进信息技术在政务、商务、公共服务和国民经济其他领域的广泛应用，扩大信息消费。策应上海世博会，加快旅游资源开发。大力发展教育、文化产业，积极发展会计、法律、科技、资产评估、咨询等中介服务业。注重运用现代经营方式和服务技术，改造和发展传统服务业。落实服务业发展引导资金，支持重点项目建设。搞好全国第三产业普查。认真做好财税金融工作。积极培植财源，营造依法纳税的社会环境，确保财政收入稳定增长。增加有效信贷投入，支持和促进经济社会发展。

以中心城市建设为龙头，继续加快城市化进程。围绕“建设大城市、塑造新形象”的目标，进一步提高中心城市规划、建设、经营、管理水平。在城市规划上，坚持以人为本原则，立足城市可持续发展，注重塑造城市特色，彰显城市文化内涵；在城市建设上，继续扩大规模，改善环境，完善功能，提升品位，重点实施第一污水处理厂、东风路二期、港区向阳路北延、凤凰河建设、稻河整治以及坡子街河滨广场和泰高路、328国道、城区街景绿化等城市基础设施工程，加快推进坡子街商城、新纪元商业广场、五一路和高港金港路商业街以及府南住宅小区等重点项目的开发，启动东城河旅游风光带的规划和建设；在城市经营上，进一步放开投资领域，精心运作土地资产，最大限度地发挥市场机制的作用；在城市管理上，积极推进综合执法，切实解决长期存在的多头执法、职责交叉、重复处罚等问题。进一步抓好县级市城区和重点中心镇建设。贯彻适度超前的原则，加大城乡基础设施建设力度。组织实施江平线靖江段、沿江公路泰州段、宁通高速泰州互通及接线、高兴东公路兴化城区过境段和宁启铁路泰州段等重点交通工程，做好兴泰公路北延的开工准备，加快地方一般公路建设，抓好经济薄弱村通村公路建设。完善信息和电力基础设施。继续抓好以河道疏浚、水环境综合整治为重点的农田水利基本建设和防汛防旱工程，完成泰东河拓浚工程年度计划。

以扩大就业再就业和完善社会保障体系为基础，努力改善人民生活。按照“强化载体、拓展空间、落实政策、优化服务”的要求，扎实做好就业再就业工作。主动适应吸纳就业载体的变化，把发展社区服务业和私营个体经济作为扩大就业再就业的主攻方向；进一步完善政策公示、联合办公、再就业绿卡和检查通报四项制度，确保国家和省、市出台的税费减免、财政投入、小额贷款、就业援助等各项政策落实到位；加强劳动力市场建设，建立健全就业和社会保障服务平台。继续做好“两个确保”和“三条保障线”衔接工作，完善养老、医疗、失业、工伤、生育保险和城市居民最低生活保障制度，重点在扩面征缴、基金筹措、管理服务、多层次保障等方面下功夫。

以三个文明协调发展为目标，加强社会主义政治文明和精神文明建设。自觉接受人大及其常委会的法律监督和工作监督，积极支持人民政协履行政治协商、民主监督、参政议政职能。主动向民主党派、工商联及工会、共青团、妇联等人民团体通报工作情况，听取意见和建议。健全村民自治，完善城市居民自治，建设管理有序、文明祥和的新型社区，坚持职工代表大会和其他形式的企事业单位民主管理制度，保障职工的合法权益。坚持依法行政，强化行政执法监督，规范行政执法行为。完成“四五”普法年度计划，提高公务员特别是领导干部的法制观念和依法办事能力。认真落实信访工作责任制，努力为群众排忧解难。深入开展“五星级”安全文明创建活动，加强矛盾纠纷排查调处工作，加大严打整治斗争力度，防范和惩治法轮功等邪教组织，遏制社会丑恶现象。高度重视安全生产，严防重特大事故发生。

广泛开展党的十六大精神学习教育活动，坚持用邓小平理论和“三个代表”重要思想武装干部、教育群众。继续贯彻《公民道德建设实施纲要》，深入开展文明城市创建活动。全面推进素质教育，提高教育质量。完善“以县为主”的农村义务教育管理体制，基本完成农村中小学布局调整，积极发展高中阶段教育，加强职业技术教育和培训，努力提高高等教育办学水平。鼓励民办教育发展。建成市莲花学校新校区和市青少年校外活动中心。深入实施科技“四大工程”，加快市高新技术创新中心建设，大力发展民营科技企业，办好市第四届科技洽谈会。重视人才引进、培养和使用，构筑人才高地。培育和繁荣文化市场，重视社区文化建设。开工建设市博物馆新馆和档案馆。实施古民居保护，修缮南山寺，启动城隍庙保护工程。积极推行大病统筹、医疗保险等合作医疗制度，提高农村卫生水平。进一步强化药品质量管理，保证人民群众用药安全。实施全民健身计划纲要，加快体育中心建设。积极发展广播电视、新闻、统计、审计、气象、地震、人防、科普、史志档案事业，做好民族、宗教、妇女儿童、老龄和残疾人工作。认真贯彻计划生育、保护环境和保护资源的基本国策，全面推进计划生育改革和优质服务，加强生态环境保护和建设，合理开发和节约使用土地等各种自然资源。加强国防教育，做好双拥、民兵预备役和征兵工作。

以提高行政水平和服务质量为主线，进一步抓好政府自身建设。面对新形势、新任务，大兴勤奋学习之风、调查研究之风、开拓创新之风、联系群众之风和艰苦奋斗之风，把政府自身建设提高到一个新水平。认真抓好公务员的理论和业务学习培训，提高队伍整体素质。坚持与时俱进，大胆开拓创新，勇于接受新理论、新观点、新方法，善于把握形势变化的新趋势、总结基层和群众的新实践、借鉴外地的新经验，坚持加快发展、富民强市不动摇、不松劲，坚持解放思想、团结拼搏不争论、不内耗，坚持科学决策不浮夸、不浮躁，努力增强领导工作的预见性、原则性、科学性和创造性。以深化软环境建设为抓手，以行政审批制度改革为突破口，大力推进政府管理体制和管理方式创新，进一步增强服务意识，提高机关服务水平。牢记全心全意为人民服务的根本宗旨，做到思想上尊重群众，感情上贴近群众，行动上深入群众，工作上依靠群众，时刻把群众的安危冷暖挂在心上。着力解决老红军、老复员军人、伤残军人、烈属等重点优扶对象和劳动模范、先进工作者以及农村五保户的实际困难，认真落实对义务兵家属的优待政策，继续做好转业复员退伍军人安置工作。重点关心和帮助困难企业和贫困地区的群众，继续推进法律援助，深入开展机关干部挂钩帮扶城市贫困户活动，扎实抓好“南户北村”挂扶工作。继续为全市人民办20件实事，为市区人民办10件实事。牢固树立长期艰苦奋斗的思想，脚踏实地，埋头苦干，不畏艰难，奋力拼搏。大力弘扬艰苦朴素、勤俭节约的精神，坚决不搞脱离实际的各种“形象工程”、“政绩工程”，坚决压缩会议费、招待费、出国考察费等一般性支出，坚决减少缺乏实际意义的参观考察活动。认真落实廉政建设责任制，扎实抓好领导干部廉洁自律工作，继续加大违纪违法案件查处力度，深入开展纠风和专项治理，进一步从源头上防范和治理腐败，树立执政为民、清正廉洁的良好形象。

各位代表，新的形势催人奋进，新的事业任重道远。让我们高举邓小平理论伟大旗帜，全面贯彻“三个代表”重要思想，在以胡锦涛同志为总书记的党中央领导下，解放思想、实事求是，与时俱进、开拓创新，沿着党的十六大指引的道路奋勇前进，为全面建设小康社会，加快“富民强市，快速崛起”进程作出新的更大的贡献！

.
